# Prognostic role of the 2MACE score in older patients with atrial fibrillation

**DOI:** 10.1007/s11739-025-04177-x

**Published:** 2026-01-22

**Authors:** Giuseppe Armentaro, Giulia Crudo, Mario Daidone, Danilo Menichelli, Matteo Bortoluzzi, Carlo Alberto Pastura, Marcello Divino, Giandomenico Severini, Egidio Imbalzano, Pasquale Pignatelli, Francesco Andreozzi, Daniele Pastori, Antonino Tuttolomondo, Angela Sciacqua

**Affiliations:** 1 Geriatrics Division, Renato Dulbecco” University Hospital of Catanzaro, 88100 Catanzaro, Italy; 2 Department of Medical and Surgical Sciences, University Magna Græcia of Catanzaro, 88100 Catanzaro, Italy; 3 Internal Medicine and Stroke Care Ward, Department of Health Promotion, Mother and Child Care, Internal Medicine and Medical Specialties, University of Palermo, Palermo, Italy; 4 Department of Clinical Internal, Anesthesiological and Cardiovascular Sciences, Sapienza University of Rome, Viale del Policlinico 155, 00161 Rome, Italy; 5 Department of Clinical and Experimental Medicine, University of Messina, Messina, Italy; 6 IRCCS Neuromed, Località Camerelle, 86077 Pozzilli, IS Italy

**Keywords:** Atrial fibrillation, 2MACE score, Elderly, DOACs, VKAs

## Abstract

Atrial fibrillation (AF) is the most common arrhythmia in the elderly and is associated with an increased risk of cardiovascular events (CVE); indeed, a percentage of patients develop CVE during oral anticoagulation. The 2MACE score is a clinical score used to predict the risk of MACE in patients with AF, but not in elderly patients with AF. Therefore, the purpose of this work is to evaluate the possible predictive value of the 2MACE score on MACE onset, in a cohort of elderly patients with non-valvular AF and several comorbidities for long-term follow-up. 1005 elderly with non-valvular AF were enrolled (76.4 ± 6.1 years; 291 on VKAs and 714 on DOACs) and stratified according to the median value of 2MACE score. MACE (non-fatal ischemic stroke, non-fatal myocardial infarction, and cardiovascular death) and all-cause mortality occurrence were evaluated during a mean follow-up of 6.2 years. In this multicenter retrospective observational study, a 2MACE score ≥ 4 pt (taken as a dichotomous value) (HR 4.38, 95% CI 3.46–5.55; *p* < 0.0001) was associated with a 4.3-fold increased risk of MACE, with modest discriminatory ability (AUC: 0.65; standard error: 0.018; 95% CI, 0.62–0.69). Furthermore, the presence of MMSE score < 24 pt (HR 1.27, 95% CI 1.03–1.56, *p* = 0.026) was associated with a 27% increased risk of MACE. In contrast, DOACs and SGLT2-inhibitor were associated with a 55% (HR 0.45, 95% CI 0.36–0.55, *p* < 0.0001) and 46% (HR 0.54, 95% CI 0.41–0.71, *p* < 0.0001) lower risk of MACE, respectively. Elderly patients with non-valvular AF whot exhibit a 2MACE score ≥ 4 pt show a higher risk of MACE and all-cause mortality.

**Supplementary Information:**

The online version contains supplementary material available at 10.1007/s11739-025-04177-x.

## Introduction

Atrial fibrillation (AF) is the most common supraventricular cardiac arrhythmia in older patients with a prevalence of 5%, and its prevalence is expected to increase 2.3-fold in the future [1, 2]. AF is associated with several cardiovascular and non-cardiovascular comorbidities, such as heart failure (HF), chronic obstructive pulmonary disease (COPD), and chronic kidney disease (CKD). Of interest, AF is associated with cognitive impairment (CoI), dementia, and increased mortality, thus being a sign of significant negative outcomes for patients, social health, and the healthcare economy [3].

Prevention of arrhythmia‐related thromboembolic risk, including the risk of stroke and peripheral thromboembolism, is an important therapeutic goal in this patient population [4]. Thromboprophylaxis is based on oral anticoagulation by direct oral anticoagulants (DOACs) or in patients not eligible for DOACs by vitamin-K antagonists (VKAs). [1, 5–7]. Older patients are exposed to an increased risk of cerebral and peripheral ischemic events; moreover, considering several comorbidities and polypharmacy, it is exposed to an increased risk of drug interactions and bleeding related to oral anticoagulant therapy [8]. These aspects often result in an underdosing of anticoagulant therapy, which exposes the elderly patient to an additional risk of ischemic events [9].

Cardiovascular (CV) risk reduction is a pivotal issue in managing patients with AF, as currently the risk of cardiac complications exceeds that of thromboembolism [10]. In this regard, a simple clinical risk score, namely 2MACE score, for predicting major adverse cardiovascular events (MACE) in patients with AF has been recently developed and validated. The 2MACE score includes metabolic syndrome, age of 75 years or older, history of myocardial infarction or revascularization, congestive HF, and history of thromboembolism [11]. In particular, a 2MACE score ≥ 3 pt identifies patients with AF at high residual risk of cardiovascular events.

Several studies confirmed the usefulness of the score for the prediction of MACE and mortality [12–14], but few data on elderly population do exist.

This is very important, because despite optimal management of thromboembolic and hemorrhagic risk according to CHA_2_DS_2_VASc and HAS-BLED score, a percentage of patients develop CV events during oral anticoagulation. Therefore, the purpose of this work is to evaluate the possible predictive value of the 2MACE score on MACE onset, in a cohort of elderly patients with non-valvular AF and several comorbidities for long-term follow-up.

## Methods

### Study setting, design, and population

In this multicenter observational retrospective study, 1005 consecutive outpatients with non-valvular AF were enrolled, referred to the Geriatrics Department, “Magna Graecia” University of Catanzaro (Italy) and department of promoting Health, Maternal-infant, Excellence and Internal and Specialized Medicine (ProMISE) G. D’Alessandro, University of Palermo (Italy). Inclusion criteria were non-valvular AF, age ≥ 65 years, and availability of at least 1 year of follow-up. Exclusion criteria were valvular diseases (mechanical prosthetic heart valves or moderate-severe mitral stenosis), already had cardiovascular events (MACEs) or autoimmune systemic diseases, active cancer, and liver failure (e.g., cirrhosis). The 2MACE score comprises 2 points each for metabolic syndrome and age equal to or greater than 75 years, and 1 point each for MI/revascularization, congestive heart failure (ejection fraction ≤ 40%), and thromboembolism (stroke/transient ischemic attack [TIA]), thus ranging from 0 to 7 [11].

The local Ethical Committee: Comitato Etico Regione Calabria “Area Centro” (protocol number 2012.63 del 17/10/2012) and Comitato Etico Regione Sicilia “Palermo 1” (protocol number 2023.04 del 19/04/2023) approved the protocol. The procedures used in this study adhere to the tenets of the Declaration of Helsinki.

### Clinical variables at baseline and follow-up

At enrollment and during the follow-up, all patients underwent a comprehensive medical history, physical examination, and routine electrocardiography as well as took measurements of anthropometric and vital variables such as weight, height, body mass index, systolic and diastolic blood pressure, and heart rate. Relevant comorbidities and the number and type of drug therapies were also recorded. The risk of ischemic stroke and bleeding was assessed using validated tools for AF patients (CHA_2_DS_2_VASc score e HAS-BLED score). The follow-up lasted 6.2 ± 3.3 years.

### Study outcomes and follow‑up

Outcomes of interest were MACEs, defined as the composite outcome of pre-specified events including cardiovascular (CV) death, MI and stroke, and its individual components. MI was defined as the development of significant Q-waves in at least two adjacent electrocardiogram leads, or at least two of the following three criteria: (1) typical prolonged severe chest pain of at least 30 min; (2) electrocardiographic changes suggestive of MI including ST-changes or T wave inversion in the electrocardiogram; (3) elevation of troponin or creatinine kinase-MB to more than the upper level of normal or, if creatinine kinase-MB was elevated at baseline, re-elevation to more than 50% increase above the previous level. Stroke was defined as an acute onset of a focal neurological deficit. CV death was defined as death due to stroke, non-central nervous system arterial embolism, pulmonary embolism, MI, hemorrhage, sudden cardiac death, pump failure, peripheral embolus or aortic dissection/rupture vascular origin lasting for 24 h or more, or that resulting in death. The details on MACE were registered, through hospital discharge letter or copy of the medical records of hospitalization, and other clinical documentation (i.e., radiology and laboratory data). In case of death, information from relatives or from the general practitioner was obtained.

### Statistical analysis

Data were expressed as mean and standard deviation (mean ± SD), for normally distributed data, as median and interquartile range (IQR) for data not normally distributed, and as number and percentage (%) for categorical variables. Student's *t*-test was performed for unpaired data for continuous variables, Wilcoxon's test for unpaired data for non-continuous variables, and *χ*2 tests for categorical variables.

At baseline, the 2MACE score was calculated for each patient, and the population was divided into two groups according to the 2MACE score median value, a group with 2MACE score < 4 pt and a group with 2MACE score ≥ 4 pt. The accuracy of the 2MACE score as a predictor of the onset of MACEs, both as a continuous and categorical variable, was evaluated by processing a receiver operating characteristic (ROC) curve. The area under the curve (AUC) described how the value of 2MACE score was associated with the onset of events. The incidence of events was calculated as the number of events per 100 patients-year. Since the follow-up was not uniform for all patients, the onset of MACE was not assessed at the same time, but a regression analysis based on the Cox proportional model was used, correcting the analysis for possible variables associated with the onset of MACEs. In particular, a Cox regression model was performed on the onset of MACEs; subsequently, the variables that were significantly (*p* < 0.05) associated with the onset of MACEs were included in a multivariate stepwise Cox regression model to calculate the hazard ratio (HR) for the independent predictors associated with the onset of MACEs. The competitive risk due to non-cardiovascular death was analyzed by calculating the sub-distribution HR (SHR) and the corresponding 95% confidence interval (CI). In addition, positive predictive value (PPV), negative predictive value (NPV), sensitivity, and specificity of the 2MACE score on onset of MACEs in the study population were calculated. The differences were considered statistically significant for *p* value < 0.05. All analyses were performed using the SPSS 20.0 statistical program for Windows (SPSS Inc., Chicago, IL, USA).

## Results

The baseline demographic, clinical,and biochemical characteristics, comorbidity, and drugs therapy of patients included in the study (*n* = 1005) are reported in Tables 1, 2. The whole population was made up of elderly patients with NVAF, with mean age 76.4 ± 6.1 years, of which 51% were females and enrolled between 2012 and 2024**.** The follow-up ended on 28-01-2024. Two follow-up visits were considered, the first follow-up after 3.7 ± 2.6 years from baseline (mid-term) and the second after 7.0 ± 3.5 years (long term). The study population suffered from several comorbidities (Table 1): 86% were affected by arterial hypertension (AH), 36% by type 2 diabetes mellitus, 32.3% were ex-smokers, 35.6% had respiratory failure/COPD, 34% had HF, and 43.58% had CKD. 714 people (71.0%) were treated with DOACs and the other 291 patients (29.0%) with VKAs. At baseline, the patients treated with DOACs were 81.4% in the 2MACE ≥ 4 group and 61.3% in the 2MACE < 4-group, *p* < 0.0001. Patients with 2MACE score ≥ 4 had a higher prevalence of AH (99.38% vs 73.21%, *p* < 0.0001), T2DM (43.62% vs 28.5%, *p* < 0.0001), being ex-smokers (40.5% vs 24.6%, *p* < 0.0001), RF/COPD (46.9% vs 25% *p* < 0.0001), HF (51.6% vs 0.175% *p* < 0.0001), and CKD (53% vs 34.68%, *p* < 0.0001).

**Table 1:** Clinical characteristics, comorbidity, and drugs therapies of the study population at baseline

	All population (*n* = 1005)			2MACE ≥ 4 (*n* = 486)		2MACE < 4 (*n* = 519)		*p*
Clinical characteristics and comorbidity								
Gender (female), n (%)	513 (51.0)			268(55.1)		245 (47.2)		0.698
Age, years	76.4 ± 6.1			79.6 ± 4.9		73.1 ± 5.5		** < 0.0001**
CHA_2_DS_2_VASc, pt	4.3 ± 1.5			5.1 ± 1.4		3.6 ± 1.2		** < 0.0001**
HAS-BLED, pt	1.4 ± 0.6			1.1 ± 0.6		1.4 ± 0.6		**0.008**
2MACE, pt	3.5 ± 1.7			4.8 ± 0.8		2.0 ± 0.9		** < 0.0001**
DOACs, n (%)	714 (71.0)			396 (81.4)		318 (61.3)		** < 0.0001**
PM/ICD, n (%)	90 (8.9)			55 (11.3)		35 (6.8)		0.058
AH, n (%)	863 (85.9)			483 (99.4)		380 (73.2)		** < 0.0001**
T2DM, n (%)	360 (35.8)			212 (43.6)		148 (28.5)		** < 0.0001**
Dyslipidemia, n (%)	382 (38.0)			205 (42.2)		177 (34.1)		0.315
Smokers, n (%)	77 (7.6)			35 (7.2)		42 (8.0)		0.258
Ex-smokers, n (%)	325 (32.3)			197 (40.5)		128 (24.6)		** < 0.0001**
Alcohol, n (%)	75 (7.5)			43 (8.8)		32 (6.2)		0.305
RF/COPD, n (%)	358 (35.6)			228 (46.9)		130 (25.0)		** < 0.0001**
Heart failure, n (%)	342 (34)			251 (51.6)		91 (17.5)		** < 0.0001**
SAS, n (%)	262 (26)			131 (26.9)		131 (25.2)		0.535
CKD, n (%)	438 (43.8)			258 (53.0)		180 (34.7)		** < 0.0001**
Liver disease, n (%)	179 (17.8)			95 (19.5)		84 (16.2)		0.515
MMSE score < 24, n (%)	281 (27.9)			158 (32.5)		123 (23.7)		0.070
Drugs therapies								
PPIs, n (%)		789 (78.5)	419 (3.9)		379 (73.0)		0.282	
Nitrates, n (%)		61 (6.0)	33 (6.8)		28 (5.4)		0.629	
ACEi/ARBs,, n (%)		768 (76.4)	414 (85.2)		354 (68.2)		**0.010**	
β-Blockers, n (%)		604 (60.0)	341 (70.2)		263 (50.7)		** < 0.0001**	
Digitalis, n (%)		148 (11.3)	96 (19.8)		52 (10.0)		** < 0.0001**	
Calcium channel blockers, n (%)		148 (14.7)	72 (14.8)		76 (14.6)		0.430	
OADs, n (%)		230 (22.9)	123 (25.3)		107 (20.6)		0.526	
Anti-arrhythmics Drugs, n (%)		167 (16.6)	60 (12.3)		107 (20.6)		** < 0.0001**	
Statins, n (%)		455 (45.3)	244 (50.2)		211 (40.6)		0.252	
MRAs, n (%)		229 (22.8)	157 (32.3)		72 (13.9)		** < 0.0001**	
ARNI, n (%)		247 (24.6)	193 (39.7)		54 (10.4)		** < 0.0001**	
SGLT2i, n (%)		218 (21.7)	142 (29.2)		76 (14.6)		** < 0.0001**	
GLP1-RAs, n (%)		5 (0.5)	1 (0.2)		4 (0.8)		0.156	

Bold highlights the statistically significant differences

2MACE, two major cardiovascular adverse events; DOACs, direct oral anticoagulants; PM, pacemaker; ICD, implantable cardioverter defibrillator; AH, arterial hypertension; T2DM, type 2 diabetes mellitus; RF, respiratory failure; COPD, chronic obstructive pulmonary disease; SAS, sleep apnea syndrome; CKD, chronic kidney disease; MMSE, Mini-Mental State Examination; PPIs, proton-pump inhibitors; ACEi, angiotensin-converting enzyme; ARBs, angiotensin receptor blockers; OADs, oral antidiabetic drugs; AADs, anti-arrhythmic drugs; MRAs, mineralocorticoid receptor antagonist; ARNI, angiotensin receptor neprilysin inhibitor; SGLT2i, sodium–glucose cotransporter 2 inhibitors; GLP1-Ras, glucagon-like peptide 1 receptor agonists

**Table 2:** Descriptive variables of the whole population at baseline

	All population (*n* = 1005)	2MACE ≥ 4 (*n* = 486)	2MACE < 4 (*n* = 519)	*p*
Clinical characteristics and comorbidity				
BMI, kg/m^2^	28.9 ± 4.5	29.4 ± 4.5	28.4 ± 4.5	** < 0.0001**
Total cholesterol mg/dl	167.0 ± 42.5	166.2 ± 42.8	167.9 ± 42.2	0.513
HDL cholesterol, mg/dl	51.1 ± 15.9	48.7 ± 14.1	53.6 ± 17.3	** < 0.0001**
LDL cholesterol, mg/dl	99.8 ± 35.2	99.6 ± 35.4	99.9 ± 35.0	0.873
Triglycerides, mg/dl	112.5 ± 53.1	117.4 ± 56.4	107.3 ± 48.7	**0.002**
Creatinine, mg/dl	1.1 ± 0.3	1.1 ± 0.3	1.0 ± 0.3	0.351
CrCl, ml/min/1.73m2	69.3 ± 24.2	66.3 ± 23.4	72.4 ± 24.7	** < 0.0001**
eGFR, ml/min	63.2 ± 18.4	61.1 ± 17.7	65.4 ± 18.8	** < 0.0001**
Na, mmol/l	140.7 ± 2.4	140.5 ± 2.4	140.9 ± 2.3	0.180
K, mmol/l	4.4 ± 0.4	4.4 ± 0.4	4.4 ± 0.4	0.594
Uric acid, mg/dl	5.6 ± 0.9	5.6 ± 1.0	5.6 ± 0.9	0.655
Hemoglobin g/dl	13.4 ± 1.5	13.3 ± 1.6	13.4 ± 1.4	0.691
PLTs, × 10^6/uL	218.7 ± 60.6	220.9 ± 61.0	216.2 ± 60.1	0.217
Albumin, g/dL	3.9 ± 0.4	3.9 ± 0.3	3.9 ± 0.4	0.307
Total bilirubin mg/dl	0.7 ± 0.3	0.8 ± 0.4	0.7 ± 0.3	0.068
Alkaline phosphatase, IU/L	88.4 ± 38.0	91.5 ± 38.0	85.2 ± 37.8	**0.008**
AST, mg/dl	20 (16–24)	20 (17–24)	20 (16–24)	0.893
ALT, mg/dl	19 (13–25)	19 (13–25)	19 (13–26)	0.459
GGT, U/l	32 (21–42)	32 (21–44)	32 (19–41)	0.117
NTproBNP, pg/ml	562 (489–1366)	632 (512–1659)	556 (485–1245)	** < 0.0001**

Bold highlights the statistically significant differences

2MACE, two major cardiovascular adverse events; BMI, body mass index; HDL, high density lipoprotein; LDL, low density lipoprotein; CrCl, creatinine clearance; e-GFR, estimated glomerular filtration rate; Na, sodium; K, potassium; PLTs, platelets; AST, aspartate aminotransferase; ALT, alanine aminotransferase; GGT, gamma-glutamyl transpeptidase; NTproBNP, pro B-type natriuretic peptide

### Cardiovascular events and non-cardiovascular mortality

**Fig. 1:**
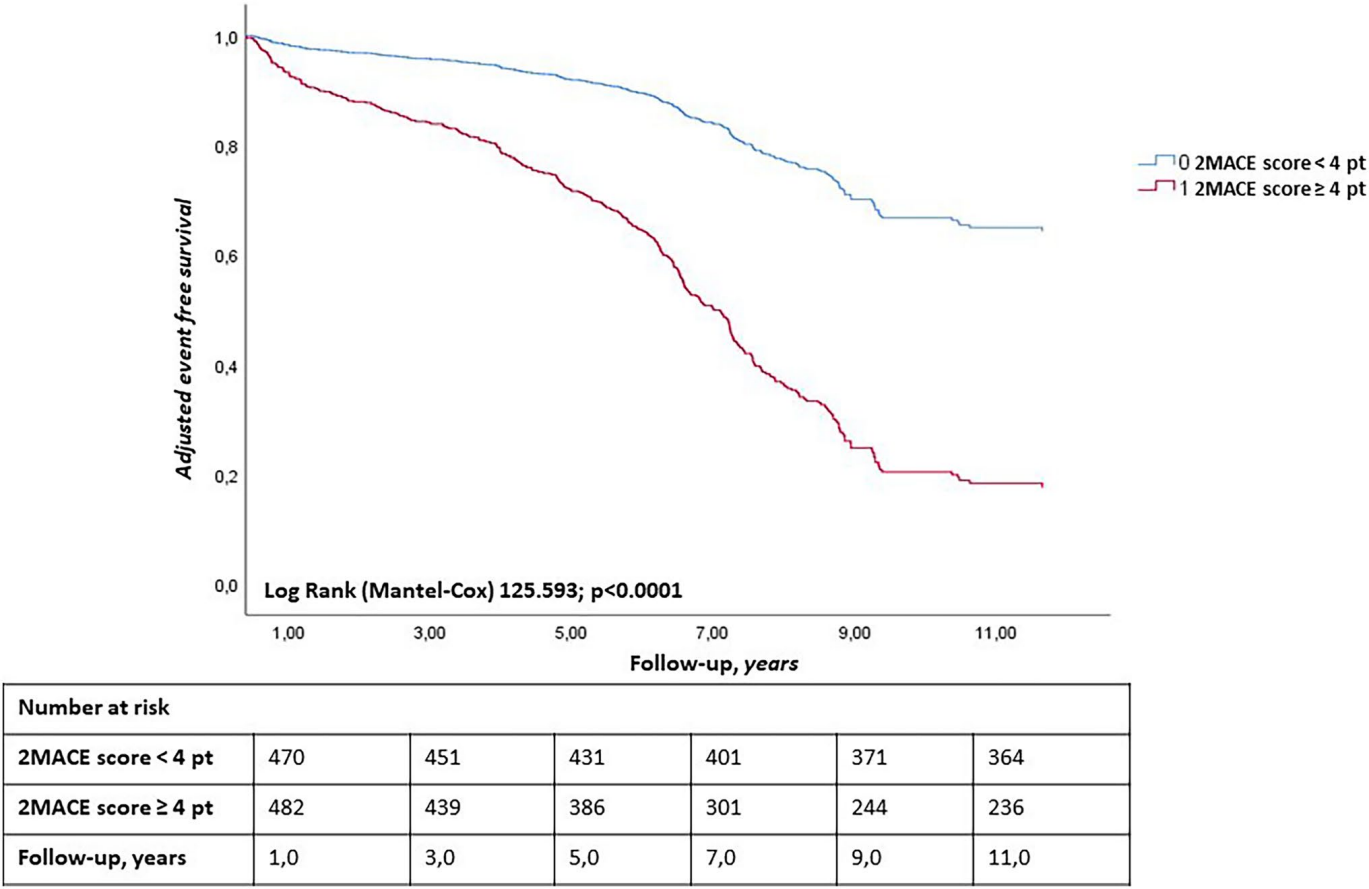
Adjusted Kaplan–Meier on onset of MACEs, according to the median value of 2MACE score in the study population. Adjusted for: DOACs therapy, MRAs, SGLT2i, MMSE ≤ 24 pt. The survival analysis, adjusted for variables that associated with the onset of MACEs, was performed using the Kaplan–Meier estimation to evaluate the onset of MACEs in patients with a 2MACE score ≥ 4 points compared to patients with 2MACE score < 4. In patients with 2MACE score < 4 pt, the observed MACEs were 122 (4.0 events/100 patient-year), while in the group with 2MACE score ≥ 4 pt 283 (8.8 events/100 patient-year) (*p* < 0.0001) (log-rank test: *p* < 0.0001). MACEs, major adverse cardiac events, 2MACE, two major cardiovascular adverse events; DOACs, non-vitamin-K oral anticoagulant; MRAs, mineralocorticoid receptor antagonist; SGLT2i, sodium–glucose cotransporter 2 inhibitors; MMSE, Mini-Mental State Examination

**Fig. 2:**
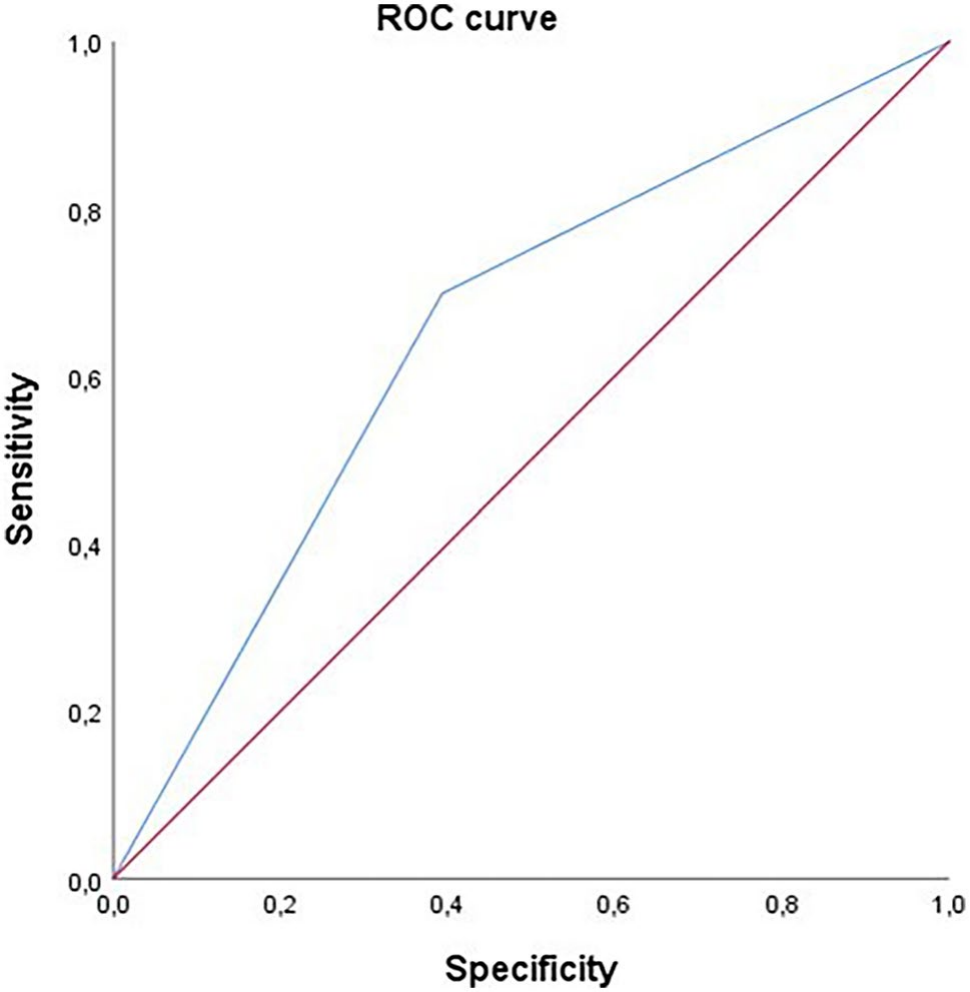
ROC curves on MACEs, according to the 2MACE score as a dichotomous variable. AUC was used to evaluate the accuracy of 2MACE score as a predictive value of MACEs occurrences as a dichotomous variable above and below the median. The ROC curve analysis demonstrated that a higher 2MACE score is associated with greater occurrence of MACEs in elderly patients with AF, considering 2MACE score as a dichotomous value the sensitivity of the test was 65% (area under the curve: 0.65; standard error: 0.018; 95% CI, 0.62–0.69). AUC, area under the curve; MACEs, major adverse cardiac events; ROC, receiver operating characteristic; 2MACE, two major cardiovascular adverse events

**Fig. 3:**
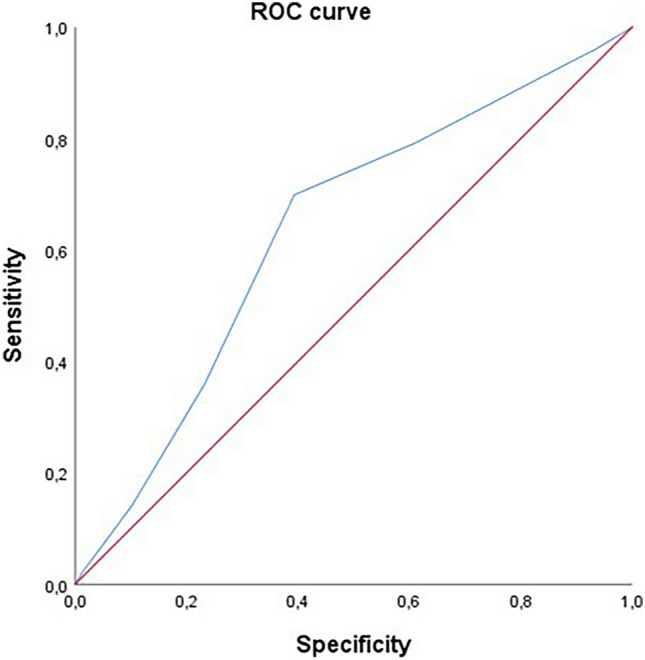
ROC curves on MACEs, according to the 2MACE score as a continuous variable. AUC was used to evaluate the accuracy of 2MACE score as a predictive value of MACEs occurrences as a continuous variable above and below the median. The ROC curve analysis demonstrated that a higher 2MACE score is associated with greater occurrence of MACEs in elderly patients with AF. Considering 2MACE score as continuous value, the sensitivity was found to be 63% (area under the curve: 0.63; standard error: 0.018; 95% CI, 0.64–0.67). AUC, area under the curve; MACEs, major adverse cardiac events; ROC, receiver operating characteristic; 2MACE, two major cardiovascular adverse events

During a mean follow-up of 6.2 years, a total of 405 MACEs were observed (6.5 events/100 patient-year). Of these, 141 were non-fatal coronary events (2.3 events/100 patient-year) and 128 were non-fatal cerebrovascular events (2.05 events/100 patient-year). In particular, in patients with 2MACE score < 4 pt, the observed MACEs were 122 (4.0 events/100 patient-year), and in the group with 2MACE score ≥ 4 pt 283 (8.8 events/100 patient-year) (*p* < 0.0001) (log-rank test: *p* < 0.0001) (Fig. 1); non-fatal coronary events were 96 in the first group (2.9 events/100 patient-year) and 45 in the second group (1.5 events/100 patient-year) (*p* < 0.0001), while non-fatal cerebrovascular events were 89 (2.8 events/100 patient-year) in the first group and 39 (1.3 events/100 patient-year) in the second group, *p* < 0.0001). A total of 136 cardiovascular deaths were observed (2.2 events/100 patient-year), 98 (3.0 events/100 patient-year) in the first group and 38 (1.3 events/100 patient-year) in the second one (*p* = 0.001) (Table 3). The total number of non-cardiovascular deaths was 52 (0.83 events/100 patient-year), 25 (0.8 events/100 patient-year) in the first group and 27 (0.9 events/100 patient-year) in the second one (*p* = 0.966) (Table 3). AUC was used to evaluate the accuracy of 2MACE score as a predictive value of MACEs occurrences, both as a dichotomous variable above and below the median (Fig. 2) and as a continuous variable (Fig. 3). The ROC curve analysis demonstrated that a higher 2MACE score was associated with greater occurrence of MACEs in elderly patients with AF; considering 2MACE score as a dichotomous value, the sensitivity of the test was 65% (area under the curve: 0.65; standard error: 0.018; 95% CI, 0.62–0.69) (Fig. 2), while considering instead the 2MACE as a continuous value, the sensitivity was found to be 63% (area under the curve: 0.63; standard error: 0.018; 95% CI, 0.64–0.67) (Fig. 3). Furthermore, this study shows that a 2MACE score ≥ 4 points is associated with an increased risk of MACEs, in the study population, with a sensitivity of 70%, specificity of 61%, PPV 55%, and NPV 75%.

**Table 3:** Incidence of MACEs, fatal and not fatal events at follow-up

	All population (*n* = 1005)	2MACE ≥ 4 (*n* = 519)	2MACE < 4 (*n* = 486)	p^*^
MACEs, n (%)	405 (6.5)	283 (8.8)	122 (4.0)	** < 0.0001**
Non-fatal coronary events, n (%)	141 (2.3)	96 (2.9)	45 (1.5)	** < 0.0001**
Cardiovascular mortality, n (%)	136 (2.2)	98 (3.0)	38 (1.3)	** < 0.0001**
Non-fatal stroke, n (%)	128 (2.05)	89 (2.8)	39 (1.3)	** < 0.0001**
Non-cardiovascular mortality, n (%)	52 (0.8)	25 (0.8)	27 (0.9)	0.597

Bold highlights the statistically significant differences

^*^Performed by Chi-square test

MACEs, major adverse cardiac events; 2MACE, two major cardiovascular adverse events

### Cox regression analysis

The survival analysis, adjusted for variables that associated with the onset of MACEs, was performed using the Kaplan–Meier estimation to evaluate the onset of MACEs in patients with a 2MACE score ≥ 4 points compared to patients with 2MACE score < 4. Figure 1 shows the survival curves for the two different subgroups. From the results of Cox regression analysis of the incidence of MACEs, a statistically significant association was found with the presence of MMSE score < 24 pt, use of DOACs, MRAs, and SGLT2i, female sex, smokers, CHA2DS2VASc, and NTproBNP levels (Table 4). The variables found to be significantly associated with the onset of MACEs in the Cox regression were included in a multivariate analysis model to define the independent predictors of MACEs (Table 5, 6). In particular, a 2MACE score ≥ 4 pt (considered as a dichotomous value) (HR 4.38, 95% CI 3.46–5.55; *p* < 0.0001) was associated with an increased risk of MACEs of 4.3-fold, and in addition the presence of MMSE score < 24 pt (HR 1.27, 95% CI 1.03–1.56, *p* = 0.026) enhanced the risk of MACEs by 27%. On the contrary, the use of DOACs (HR 0.45, 95% CI 0.36–0.55, *p* < 0.0001) was associated with a lower risk of MACEs by 55%. Finally, the use of SGLT2i (HR 0.54, 95% CI 0.41–0.71, *p* < 0.0001) was associated with a reduced risk of MACEs by 46% (Table 5). Furthermore, the 2MACE score ≥ 4 pt was associated with an increased risk of MACE (SHR 4.31; 95% CI 3.42–5.44, *p* < 0.0001) even when performing a competitive risk assessment by calculating the SHR (see Table 6). Furthermore, from the Kaplan–Meier curves, we derived the cumulative risk in the two groups during follow-up and calculated the risk ratio (Supplementary Table S1). Since the risk ratio is approximately constant over time (risk ratio of approximately 2), this means that the discriminating capacity of the 2MACE score is constant over time.

**Table 4:** Cox regression: MACEs as dependent variable

	HR	CI 95%	*p*
DOACs, yes/no	0.51	0.39–0.66	** < 0.0001**
2MACE ≥ 4pt, yes/no	4.10	2.94–5.73	** < 0.0001**
MRAs, yes/no	0.69	0.49–0.99	**0.043**
ARNI, yes/no	0.73	0.47–1.14	0.170
SGLT2i, yes/no	0.48	0.32–0.73	**0.001**
MMSE score < 24, yes/no	1.27	1.01–1.58	**0.039**
Female gender, yes/no	1.34	1.04–1.72	**0.021**
Age < 75 years, yes/no	0.96	0.69–1.33	0.792
AH, yes/no	1.34	0.93–1.97	0.120
Smokers, yes/no	1.55	1.01–2.36	**0.043**
Ex-smokers, yes/no	1.27	0.99–1.64	0.057
Alcohol user, yes/no	1.43	0.84–2.45	0.184
RF/COPD, yes/no	1.06	0.84–1.34	0.629
CHA_2_DS_2_VASc, yes/no	1.14	1.01–1.30	**0.048**
HAS-BLED, yes/no	0.89	0.65–1.22	0.484
ACEi/ARBs, yes/no	0.83	0.63–1.10	0.198
β-Blockers, yes/no	0.92	0.73–1.14	0.449
OADs, yes/no	0.80	0.59–1.08	0.148
AADs, yes/no	0.96	0.73–1.30	0.861
Statins, yes/no	0.88	0.70–1.10	0.241
LDL cholesterol, mg/dl	0.99	0.99–1.00	0.181
Triglycerides, mg/dl	1.00	0.99–1.00	0.275
CKD, yes/no	1.01	0.99–1.03	0.933
Na, mmol/l	0.99	0.95–1.04	0.752
K, mmol/l	1.05	0.78–1.42	0.721
Uric acid, mg/dl	0.96	0.86–1.06	0.404
Hemoglobin, g/dl	1.02	0.95–1.11	0.469
Albumin, g/dl	1.09	0.82–1.45	0.551
AST, UI/L	0.99	0.97–1.01	0.379
ALT, UI/L	1.00	0.99–1.01	0.794
Total bilirubin, mg/dl	0.88	0.63–1.23	0.476
Alkaline phosphatase, UI/L	0.99	0.99–1.00	0.062
GGT, UI/L	1.00	0.99–1.00	0.218
HbA1c, %	0.97	0.84–1.13	0.695
NTproBNP, pg/ml	1.09	0.81–1.29	0.886
Liver disease, yes/no	0.97	0.64–1.48	0.900
PP, mmHg	1.00	0.99–1.01	0.792

Bold highlights the statistically significant differences

MACEs, major adverse cardiac events; 2MACE, 2 major cardiovascular adverse events; VKAs, vitamin-K antagonists; DOACs, direct oral anticoagulants; MRAs, mineralocorticoid receptor antagonist; ARNI, angiotensin receptor neprilysin inhibitor; SGLT2i, sodium–glucose cotransporter 2 inhibitors; MMSE, Mini-Mental State Examination; R.F., respiratory failure; COPD, chronic obstructive pulmonary disease; ACEi, angiotensin-converting enzyme; ARBs, angiotensin receptor blockers; OADs, oral antidiabetic drugs; AADs, anti-arrhythmics drugs; LDL, low density lipoprotein; e-GFR, estimated glomerular filtration rate; CKD, chronic kidney disease; Na, sodium; K, potassium; AST, aspartate aminotransferase; ALT, alanine aminotransferase; GGT, gamma-glutamyl transpeptidase; HbA1c, glycated hemoglobin; NTproBNP, pro B-type natriuretic peptide; PP, pulse pressure

**Table 5:** Cox stepwise multivariate regression: MACEs as dependent variable, not accounting for competing risk

	HR	CI 95%	*p*
2MACE ≥ 4 pt, yes/no	4.38	3.46–5.55	** < 0.0001**
MMSE < 24 pt, yes/no	1.27	1.03–1.56	**0.026**
DOACs, yes/no	0.45	0.36–0.55	** < 0.0001**
SGLT2i, yes/no	0.54	0.41–0.71	** < 0.0001**

Bold highlights the statistically significant differences

MACEs, major adverse cardiac events; 2MACE, two major cardiovascular adverse events; MMSE, Mini-Mental State Examination; DOACs, direct oral anticoagulants; SGLT2i, sodium–glucose cotransporter 2 inhibitors

**Table 6:** Cox stepwise multivariate regression: MACEs as dependent variable, accounting for competing risk

	SHR	CI 95%	*p*
2MACE ≥ 4 pt, yes/no	4.31	3.42–5.44	** < 0.0001**
MMSE < 24 pt, yes/no	1.28	1.04–1.57	**0.016**
DOACs, yes/no	0.39	0.32–0.48	** < 0.0001**
SGLT2i, yes/no	0.66	0.53–0.81	** < 0.0001**

Bold highlights the statistically significant differences

MACEs, major adverse cardiac events; SHR, sub-distribution hazard ratio; 2MACE, two major cardiovascular adverse events; MMSE, Mini-Mental State Examination; DOACs, direct oral anticoagulants; SGLT2i, sodium–glucose cotransporter 2 inhibitors

## Discussion

In our study including elderly patients with AF, we found that the 2MACE score was significantly associated with the risk of MACEs. In particular, a 2MACE score ≥ 4 pt was associated with a 4.3-fold increased risk of MACEs.

The 2MACE score's predictive ability has been confirmed in several real-world patient cohorts, demonstrating its usefulness in clinical settings for stratifying cardiovascular risk and guiding management decisions for patients [12].

In “real-world” patients with AF, recruited from two different cohorts (Murcia AF and FANTASIIA), the 2MACE score was a good predictor of MACEs and a score ≥ 3 was used to categorize patients at “high risk” of MACEs [12].

A progressive increase in the mortality rate was observed with an increasing 2MACE score [13]. Furthermore, the predictive validity of the 2MACE score for the occurrence of MACEs in patients with AF without CAD was evaluated and may have a role in risk stratification and primary prevention of MACEs in patients with AF [14].

In addition, the 2MACE score's predictive ability was confirmed within a large international registry of AF patients, confirming the score's utility in clinical settings for identifying high-risk patients ​[15].

In an observational, multicenter, post-authorization and prospective study that involved AF patients under oral anticoagulation with rivaroxaban, after 2.5 years of follow-up, patients with 2MACE score ≥ 3 had around sixfold risk of cardiovascular death due to HF than patients with 2MACE score < 3 [15].

Age was associated with an increased risk of cardiovascular disease (CVD) [16, 17]. In this context, a good assessment of the CV risk of older patients is essential to guarantee the best therapeutic treatment. However, 2MACE score has been validated for a small cohort of patients, often in retrospective studies and limited settings, but not in the elderly population with several comorbidities and high CV risk [18].

Our work shows that the elderly population with NVAF and a 2MACE score ≥ 4 pt is associated with a 4.3-fold increased risk of MACEs (HR 4.38, 95% CI 3.46–5.55; *p* < 0.0001). However, we have no data regarding older patients, because this score has not been validated in this cohort of patients, even though there is an indication of anticoagulant therapy in patients over 80 years old, considering the fact that the risk of events is high, and the benefit of anticoagulation outweighs the risk of bleeding [19].

Recently, a prospective study was published that tried to validate a 2MACE score in a population of 25,696 patients, but the median age was 71 years (64–81), and 45% of these patients were females. The follow-up period was 3 years with scheduled visits at 6, 12, 24, and 36 months. This study has demonstrated that older patients have an increased risk of MACEs, with the 2MACE score being positively associated with an increased risk of MACEs, with a cutoff ≥ 2 (HR: 2.47, 95% CI 2.21–2.77, *p* < 0.001) [20].

Moreover, elderly patients in addition to AF suffer from several comorbidities that complicate their management, quality of life, and prognosis, including HF and cognitive impairment (CoI) [7].

As such, AF is associated with an increased risk of CoI; in fact, there are many studies that prove a significant association between AF and the accelerated development of CoI. It was also shown that AF and CoI share some risk factors and aging is the most relevant [21–23].

Nanquing Xiong et al. demonstrated that MMSE score was significantly lower in AF patients than control group and that the MMSE score was negatively associated with the CHA_2_DS_2_VASc score in patients with AF. The relationship between these two scores can be explained by increased subclinical cerebral ischemia, thus suggesting that anticoagulant therapy could be useful to reduce MACE in those patients. This study showed that patients with lower MMSE scores had a higher incidence of coronary artery disease [24].

Of interest, these data are confirmed by a recent study that demonstrated a high number of cerebral infarcts in cortical or non-cortical region by using MRI in patients with AF. Most of them had no symptoms, but they had worse cognitive functions, and this could be related to microbleeds, hypoxia, or other conditions that can be useful to prove the relationship between AF and cognitive decline [25].

Our work also showed that the presence of CoI, assessed as Mini-Mental State Examination (MMSE) < 24 pt, was associated with a 27% higher risk of MACEs (HR 1.27, 95% CI 1.03–1.56, *p* = 0.026). This is in line with data already present in the literature, where it is emphasized that the presence of cognitive decline can worsen the prognosis of AF patients.

In fact, one study conducted by Malavasi demonstrated the relationship between an MMSE < 24 and all-cause mortality (HR: 2.47, 95% CI 1.06–5.75, *p* = 0.036), thus underlying the fact that in patients with AF, CoI (MMSE < 24) is related to a worse outcome. However, the follow-up in this study was short, only 887 days, and the mean age of the population (73.4 years) was significantly lower than in our study population (76.4 years). Moreover, the number of comorbidities and the thromboembolic risk were also lower than in the population enrolled in our work (CHA_2_DS_2_VASc: 3 vs 4.3 pts) [26, 27].

In this context, the use of anticoagulants appears to be of considerable interest since, in addition to reducing the thromboembolic risk, they are associated with a reduction in the risk of cognitive decline. In particular, the use of DOACs, besides being safer than VKAs on cerebral bleeding, is associated with a lower risk of cognitive decline and dementia, as shown by several studies [28–30].

DOACs are generally safer than VKAs in the prevention and treatment of venous thromboembolism and stroke in patients affected by NVAF, thus representing a more manageable drug in preventing ischemic stroke or systemic embolism [5, 6, 9, 31].

In addition, DOACs appear to be associated with a lower incidence of major cerebrovascular events. In fact, in our study, DOACs therapy was associated with a 55% reduced risk of MACEs compared to VKAs (HR 0.45, 95% CI 0.36–0.55, *p* < 0.0001), data which are also confirmed by other studies. In fact, a propensity score-matched cohort study of 5254 OAC users with AF from the USA showed that DOACs were associated with a reduction in stroke, transient ischemic attack, or dementia compared with VKAs use (HR: 0.49, 95% CI 0.35–0.69) [32].

A large Swedish study comparing the effect of DOACs vs VKAs on ischemic or hemorrhagic stroke or dementia among low-risk individuals suggested a protective effect of DOACs, although CIs were wide (HR: 0.47, 95% CI 0.18–1.22) [33]. However, since in our study we do not have the time spent in the therapeutic range (TTR) by patients on therapy with VKAs, and that therapy with DOACs is associated with a reduced risk of MACEs, this represents a potential confounding factor, albeit in line with the results of the studies already mentioned.

Instead, in our study, the use of SGLT2i was associated with a reduced risk of MACEs by 46%.

Our study has limitations. The observational study design does not allow for definitively establishing a cause–effect relationship between the use of 2MACE score and MACEs incidence over time.

Thus, our study is purely hypothesis generating. Another limitation of the study is represented by the absence of time in the therapeutic range of the patients treated with VKAs, absence of information regarding drug therapy during follow-up, absence of data regarding evaluation of frailty, and long recruitment time. Another limitation of the study is the shorter follow-up period in the second center. In addition, we observed a suboptimal sensitivity and specificity in identifying patients at risk of MACE, despite a modest discriminatory ability of the 2MACE score (AUC = 0.65). However, the study also has strengths including the enrollment of a population usually underrepresented in clinical trials such as the elderly with several comorbidities (average age 76.4 ± 6.1). Furthermore, another strength was the long mean follow-up period (6.2 ± 3.3 years) which allowed us to optimally evaluate the validity of the score to predict the onset of MACEs in the elderly population.

Finally, patients enrolled were incident users (or new users), thus eliminating the possibility of prevalent user bias related to previous VKAs exposition. Therefore, given the aging of the general population and the burden of comorbidities, it is crucial to assess all risk factors that may be associated with an increased risk of MACEs in the elderly population to improve the prognosis and quality of life.

In conclusion, our study shows that the 2MACE score is able to predict all MACEs, even in a high-risk population such as the elderly.

Overall, the 2MACE score is a valuable instrument for assessing cardiovascular risk in elderly patients with AF. It enables healthcare providers to identify high-risk individuals and customize their treatment plans to avert adverse cardiovascular events.

## Conclusion

This study, conducted on elderly outpatients with AF and several comorbidities, showed that there is an association between a higher 2MACE score and MACEs occurrence during a long-term follow-up. In particular, a value ≥ 4 pt of 2MACE score was associated with a 4.3-fold increased risk. Notably, this association persists even after adjustment for confounding factors such as age, comorbidities, and pharmacotherapies. Present data suggest the potential usefulness of the 2MACE score as a prognostic factor, with a modest discriminatory ability, in identifying MACE in the elderly with a higher cardiovascular risk.

## Supplementary Information

Below is the link to the electronic supplementary material.

Supplementary file1 (DOCX 8 KB)

## Data Availability

The raw data supporting the conclusions of this article will be made available by the authors, without undue reservation.

## Declarations

### Code availability

Not available.
