# Supplementary material for: Prognostic role of the 2MACE score in older patients with atrial fibrillation
Source: Intern Emerg Med. 2026 Jan 22;21(2):447–57. doi: 10.1007/s11739-025-04177-x (PMC13061836; doi:10.1007/s11739-025-04177-x)
Supplement: Supplementary file 1 — Supplementary file1 (DOCX 8 KB) [file 11739_2025_4177_MOESM1_ESM.docx]

**Supplementary table 1 - Risk Ratio of the study population according 2MACE score**

|  | **2MACE score <4 pt** | **2MACE score≥4 pt** |  |
| --- | --- | --- | --- |
| **Time** | **Risk** | **Risk** | **Risk Ratio** |
| 1 year | 3.4 | 7.5 | 2.2 |
| 2 years | 4.9 | 13.2 | 2.7 |
| 3 years | 7.5 | 15.9 | 2.1 |
| 4 years | 10.3 | 20.3 | 2.0 |
| 5 years | 12.2 | 27.1 | 2.2 |
| 6 years | 14.3 | 34.7 | 2.4 |
| 7 years | 20.6 | 47.7 | 2.3 |
| 8 years | 27.2 | 62.7 | 2.3 |
